# Identification and Characterization of an HtrA Sheddase Produced by *Coxiella burnetii*

**DOI:** 10.3390/ijms241310904

**Published:** 2023-06-30

**Authors:** Ikram Omar Osman, Aurelia Caputo, Lucile Pinault, Jean-Louis Mege, Anthony Levasseur, Christian A. Devaux

**Affiliations:** 1 Microbes Evolution Phylogeny and Infection (MEPHI) Laboratory, Aix-Marseille University, Institut de Recherche Pour le Développement (IRD), Assistance Publique Hôpitaux de Marseille (APHM), Institut Hospitalo-Universitaire (IHU)–Méditerranée Infection, 13005 Marseille, France; ikram-ayan@hotmail.fr (I.O.O.);; 2 Laboratory of Immunology, Assitance Publique-Hôpitaux de Marseille (APHM), 13005 Marseille, France; 3 Centre National de la Recherche Scientifique (CNRS), 13009 Marseille, France

**Keywords:** Q fever, *Coxiella burnetii*, HtrA, sheddase, E-cadherin

## Abstract

Having previously shown that soluble E-cadherin (sE-cad) is found in sera of Q fever patients and that infection of BeWo cells by *C. burnetii* leads to modulation of the E-cad/β-cat pathway, our purpose was to identify which sheddase(s) might catalyze the cleavage of E-cad. Here, we searched for a direct mechanism of cleavage initiated by the bacterium itself, assuming the possible synthesis of a sheddase encoded in the genome of *C. burnetii* or an indirect mechanism based on the activation of a human sheddase. Using a straightforward bioinformatics approach to scan the complete genomes of four laboratory strains of *C. burnetii*, we demonstrate that *C. burnetii* encodes a 451 amino acid sheddase (CbHtrA) belonging to the HtrA family that is differently expressed according to the bacterial virulence. An artificial CbHtrA gene (CoxbHtrA) was expressed, and the CoxbHtrA recombinant protein was found to have sheddase activity. We also found evidence that the *C. burnetii* infection triggers an over-induction of the human HuHtrA gene expression. Finally, we demonstrate that cleavage of E-cad by CoxbHtrA on macrophages-THP-1 cells leads to an M2 polarization of the target cells and the induction of their secretion of IL-10, which “disarms” the target cells and improves *C. burnetii* replication. Taken together, these results demonstrate that the genome of *C*. *burnetii* encodes a functional HtrA sheddase and establishes a link between the HtrA sheddase-induced cleavage of E-cad, the M2 polarization of the target cells and their secretion of IL-10, and the intracellular replication of *C. burnetii*.

## 1. Introduction

*C. burnetii* is an obligate intracellular pathogen that contaminates the host in the upper airways and is responsible for Q fever in humans [1]. The bacteria are phagocytosed by pulmonary alveolar macrophages (myeloid cells), and *C. burnetii* replicates in late phagosomes [2]. In non-myeloid cells, *C. burnetii* replication occurs in an acidic phagolysosomal vacuole named CCV (for *C. burnetii-*containing vacuole), which requires a type IV secretion system (T4SS) to be fully established [3]. In CCV, the bacteria differentiate into the large cell variant and replicate; then, the bacteria differentiate back into the infectious small cell variant and lyse the cell [4]. Two to four weeks after bacterial exposure, the infected humans frequently present an acute fever (named Q fever). The symptoms of acute Q fever usually resolve spontaneously in a few weeks except in the 5% of patients who develop persistent Q fever [1,5]. Persistent Q fever is sometimes associated with endocarditis complications, while interstitial lung diseases, persistent granulomatous hepatitis, cholecystis, and hemophagocytic syndrome have also been reported [6]. Persistent Q fever patients have been considered to be at a higher risk of developing non-Hodgkin’s lymphoma (NHL) [6,7,8,9]. However, this last observation is controversial, and other studies of Q fever patients have not confirmed the link between Q fever and NHL [10,11]. If such a link did exist, it would be very rare and would result from a complex and long process of B-cell reprogramming.

Increasing evidence indicates that epithelial cadherin (E-cad) can play a major role during the development of infectious diseases. During the human host invasion by bacteria, overexpression of proteases called sheddases can lead to intercellular adhesion molecule cleavage and tissue destruction, leading to the transmigration of bacteria [12,13]. *Listeria monocytogenes* binds to intestinal epithelial cells through E-cad [14]. *Helicobacter pylori* and *Chlamydia trachomatis* infection was also found to be associated with the methylation of the *CDH1* promoter and down-regulation of E-cad [15,16]. High levels of sE-cad were reported in the biological fluids of patients with gastric adenocarcinoma induced by *Helicobacter pylori* and colon cancer induced by *Bacteroides fragilis* or *Streptococcus gallolyticus* [17,18,19,20]. Changes in surface expression levels of adhesion molecules constitute a common mechanism for modulating cell-to-cell contact and cell reprogramming [21]. Tissue migration of immune-competent cells requires the sheddase-mediated degradation of transmembrane cell adhesion molecules (CAM) [22], whereas an overexpression of CAM delays cell migration [23]. Among cadherins, the E-cad, a 120 kDa cell-surface CAM, is known for its role in the cell-to-cell interaction crucial to ensuring the integrity of epithelia and cell polarity. Moreover, this trans-membrane protein behaves as a signaling molecule through its intra-cytoplasmic tail, which binds second messengers (β-catenin), thereby playing a role in gene control, cell activation, division, differentiation, and/or invasion [24]. Cell reprogramming can be triggered by the release of soluble E-cadherin (sE-cad), an 80 kDa fragment generated from the proteolysis of the extracellular domain of E-cad [25]. Elevated sE-cad concentrations in patients’ fluids were found to be associated with a progression towards multiple myeloma and solid tumors [26].

We previously reported high levels of sE-cad in the sera of patients infected by *C. burnetiid* [27]. We speculated that if there is a link between persistent Q fever and rare cases of NHL, one of the first steps towards NHL progression might be the overexpression of E-cad on monocytes and the decrease in E-cad surface expression on the E-cad+CD20+ B-cells subpopulation of PBMCs (less than 1% of the B cells) from *C. burnetii*-infected patients [27]. In addition, because of the rapidly growing list of bacteria that mediate the proteolysis of E-cad to alter the integrity of epithelial tissues and initiate an invasion process [28], we also postulated that E-cad cleavage may be a step towards *C. burnetii* invasion of the host during Q fever. Thus, the question arises about the nature of the molecule(s) catalyzing the cleavage of E-cad in Q fever patients. Cleavage of the extracellular domain of E-cad has been reported to be achieved by a growing family of sheddases. Although elevated concentrations of the MMP-9 sheddase in the sera of patients with acute Q fever and MMP-7 sheddase in the sera of patients with persistent Q fever were previously reported [29,30], it is tempting to postulate that prokaryotic sheddases encoded by *C. burnetii* could increase E-cad proteolysis [31], as previously reported for several bacteria [25,32,33].

We report here the results of an in silico search for *C. burnetii* genes and proteins having homologies with known sheddases and provide the first direct evidence that *C. burnetii* can synthesize its own functional HtrA sheddase. We discuss the possible implications of these results on the replication cycle of *C. burnetii.*

## 2. Results

### 2.1. In Silico Search for a Hypothetical Sheddase in the Genome of C. burnetii

The proteolytic cleavage of the E-cad and release of sE-cad observed in BeWo cells after *C. burnetii* infection [31] suggest that the bacterial infection is accompanied by expression of sheddase(s). Since sheddases may be either of cellular origin or encoded by infectious pathogens, we first focused our attention on investigation of a possible bacterial origin of the sheddase involved in releasing the sE-cad.

A bioinformatic strategy was set up in order to search for genes potentially coding sheddases in the genome of *C. burnetii*. First, the genes coding for 22 sheddases were blasted against the full genome (1481 genes screened) from four strains of *C. burnetii* (RSA 493 Nine Mile; NL3262 Netherland, Z3055 clone related to the NL3262 strain and Cb175 Guiana strain) in search of homologous sequences. Because this early screening was unsuccessful, a similar investigation was conducted at the protein level using the sequences from the following: MMP2, -3, -7, -9, and -14; ADAM10 and -15; PITX1 pituitary homeobox; plasminogen; the three isoforms of KLK-7; cathepsin; the two isoforms of DCST1 E3 ubiquitin protein ligase; the three isoforms of EFNA4; BFT; *B. fragilis* toxin; and HtrA, the *Salmonella typhimurium* high-temperature-requirement protein A. These protein sequences of sheddases were aligned against the different open-reading frames (hypothetical gene products) of *C. burnetii*. Of the 22 sheddases tested, only 3 human sheddases (HuMMP-9; HuADAM-15; and HuMMP-3), 1 parasite sheddase (rhomboid), and 2 sheddases of bacterial origin (subtilase and HtrA) revealed significant alignment with hypothetical gene products from *C. burnetii*. Except for the bacterial HtrA, the other protein sequences matched with only a short portion of a known or hypothetical protein from *C. burnetii* (Table 1).

Regarding the similarities with sheddases of human (Hu) origin, THREE strains of *C. burnetii* (RSA 493 Nine Mile, NL3262 Netherland, and Z3055) expressed a 79-amino-acid sequence presenting 25% identity and 79% coverage with HuMMP-9 (expected (e)-value of 10^−3^). This sequence matched with amino acids 539 to 660 of HuMMP-9 (a protein composed of 707 amino acid residues), a region corresponding to a hemopexin domain. The Guiana strain Cb175 (known to lack genes compared to the other strains) lacks expression of this *C. burnetii* (Cb) MMP-9-like protein sequence. In contrast, the Cb175 strain expresses a protein presenting 30% identity and 50% coverage with HuADAM-15 (e-value 7 × 10^−4^) that is not found within the genome of the other *C. burnetii* strains studied. Finally, all strains of *C. burnetii* encode a protein sequence showing 28–30% identity with the 1/3 NH-2 terminal region of the HuMMP-3 metalloprotease (a protein composed of 477 amino acid residues). Regarding the parasite and bacterial sheddases, sequence identity was found for rhomboid, subtilase, and HtrA.

The blasting search in the *NCBI* database revealed that the CbMMP-3-like sequence corresponds to the nuclear transport factor 2 protein and that the CbMMP-9-like sequence corresponds to the L28 protein of the large 50S ribosomal subunit of *C. burnetii* (Table 2). Because the CbMMP-3 like/nuclear transport factor 2 protein shows unexpected similarities with HuMMP-3 in the region that corresponds to the catalytic domain of HuMMP-3 and shares some sequence similarities with the BFT toxin bacterial sheddase, we performed a reverse BLAST that led us to the conclusion that it is very unlikely that such a molecule might exhibit sheddase activity. Using the same approach, we also eliminated CbMMP-9 like/L28 protein and CbADAM-15-like sequences as possible candidates for sheddase activity. Two additional hypothetical *C. burnetii* proteins with either Rif1 or OmpA domains emerged from the in silico screening. However, none of those molecules met the criteria for a possible sheddase.

Thus, the only protein that met our screening criteria was the *C. burnetii* (Cb) HtrA-like molecule (CbHtrA). Interestingly, the four strains of *C. burnetii* were found to express this hypothetical protein showing 42% identity and 100% coverage with the bacterial HtrA protein of *Salmonella typhimurium* (e-value: 2 × 10^−112^) (Table 2), representing the best match among the studied sheddases. Among the primary hits, the in silico analysis indicated that the CbHtrA was one of three hits containing a signal peptide compatible with secretion.

### 2.2. Identification of an HtrA-like Sheddase Produced by Coxiella Burnetii

The search for the protein sequence identity of HtrA from *Salmonella typhimurium* in *C. burnetii* revealed that the four strains of *C. burnetii* express a hypothetical protein showing 42% sequence identity and 100% coverage with HtrA (e-value: 2 × 10^−112^). HtrA serine proteases are frequently found in bacteria and have already been described as involved in the catalytic cleavage of E-cad during the human host invasion. These molecules are located in the periplasm and have both protease and chaperon functions. The protease activity is turned on to its active form by heat shock.

**Figure 1:**
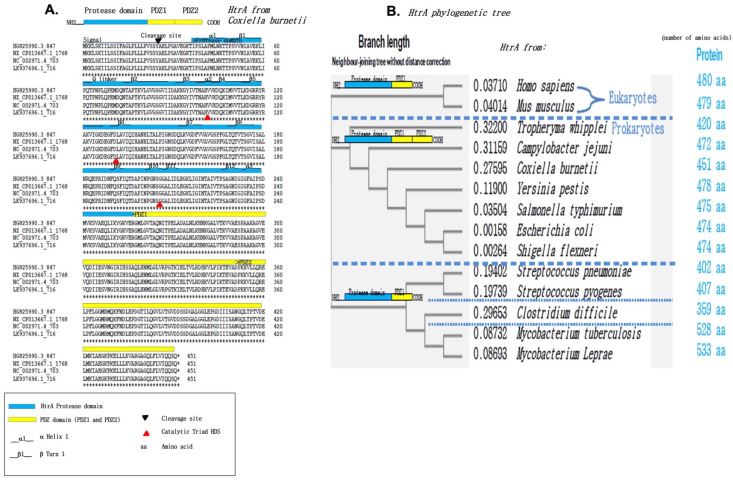
Characterization of heat-shock-induced serine protease HtrA from *Coxiella burnetii*. (**A**) CLUSTAL sequence alignments of the cbHtrA hypothetical protein from *C. burnetii* Cb175 Guiana strain (Accession nb: HG825990.3), NL3262 Netherland strain (Accession nb: NZ_CP013667.1), RSA 493 Montana USA strain Nine Mile (Accession nb: NC_002971.4), and Z3055 strain (Accession nb: LK937696). The cbHtrA hypothetical protein in the different strains of *C. burnetii* contains 451 amino acid residues and shows 100% amino acid identity from one strain to another. The assignment of signal peptide, serine protease domain (that includes the catalytic triad HDS), and PDZ (protein-protein interaction) domains is, according to [34], the catalytic triad of H, D, and S, described as essential to function [33,35], which appears to be conserved in the hypothetical CbHtrA. “*” indicates the preservation of the nucleotide sequence. (**B**) The HtrA phylogenetic neighbor-joining tree was built using the Clustal Omega program and the proteins’ best match for known HtrA from eukaryotes (human and mouse) and prokaryotes. The length of HtrA proteases varies from one species to another (from 359 amino acids to 533 amino acids). Some prokaryotic HtrA contain two PDZ domains, while others only contain one PDZ domain. The proteins’ best match to CbHtrA is with a group of HtrA proteases from prokaryotes including *Campylobacter jejuni*, *Yersinia pestis*, *Salmonella typhimurium*, and *Escherichia coli*.

The Clustal Omega multiple-sequence alignment of four strains of *C. burnetiid*—the Cb175 strain, the 3262 strain, the RSA 493 strain, and the Z3055 strain—demonstrates that the hypothetical CbHtrA sequence of 451 amino acid residues is 100% identical in the four bacterial strains (Figure 1A). This hypothetical CbHtrA is characterized by a common organization that includes a signal peptide, a serine protease domain, and two PDZ (postsynaptic density protein 95, Drosophila disc large tumor suppressor, and Zonula occludens-1 protein domain) domains. CbHtrA exhibits high sequence identity with the HtrA from other bacterial species such as *Tropheryma whipplei*, *Campilobacter jejuni*, *Yersinia pestis*, *Salmonella typhimurium*, enteropathogenic *Escherichia coli*, and *Shigella flexneri,* while this sequence is genetically more distant from the HtrA sequences of Mycobacteria and from the HtrA sequences of eukaryotes characterized by a single PDZ domain (Figure 1B). Within the serine protease domain, the catalytic triad of H, D, and S, described as essential to function, appears conserved in the hypothetical CbHtrA. Altogether, these results suggest that a secreted form of the CbHtrA protein could be functionally active.

### 2.3. Transcription of the CbHtrA-like Gene in C. burnetii-Infected Cells

**Figure 2:**
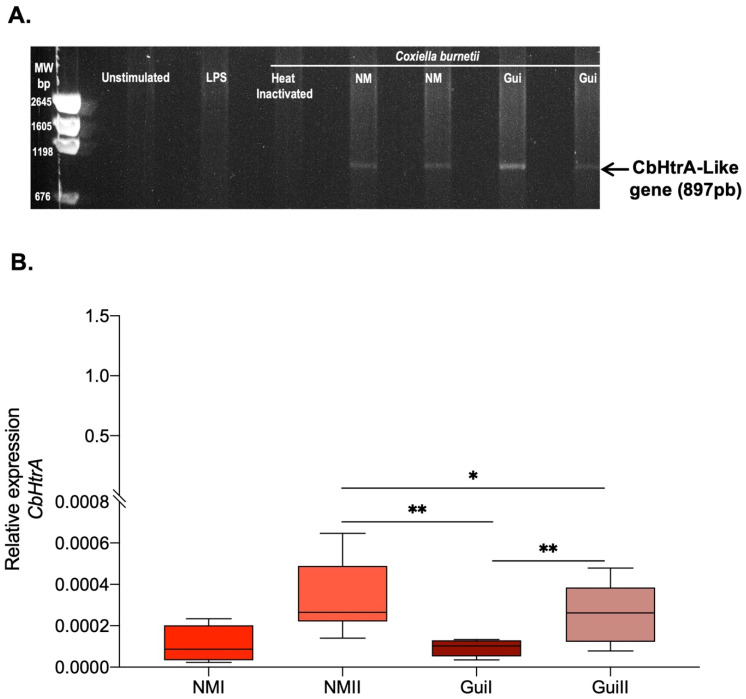
Expression of CbHtrA in BeWo cells infected by *C. burnetii* (n = 6). (**A**) RT-PCR amplification on BeWo cells uninfected or infected with the bacterium. Heat-inactivated *C. burnetii*, *E. coli* LPS, or unstimulated BeWo cells were used as controls to demonstrate the lack of expression of the *CbHtrA* using the oligonucleotide primers specific to the *CbHtrA*-like gene. (**B**) The qRT-PCR analysis of the *CbHtrA* gene expression in BeWo cells infected by the Nine Mile (NM) and the more aggressive Guiana (Gui) strain of *C. burnetii* using primers specific to the CbHtrA. The expression level of investigated genes is illustrated as ± SEM of the relative expression (RE = 2^−ΔCT^, where Δct = [Ct_target_ − Ct_actin_]) and compared using the Mann–Whitney U-test. For *p*-value < 0.05: symbol *; *p*-value < 0.01: symbol **.

The human BeWo cells were previously reported to be susceptible to *C. burnetii* infection and to express high amounts of E-cad at their surface [31],which makes this cell line an interesting and practical cellular model for studying the expression of the CbHtrA-like gene in these cells once infected with *C. burnetii*. The presence of the CbHtrA gene in the genome of the Nine Mile (NM) and Guiana (Gui) strains of *C. burnetii* was demonstrated by PCR analysis using CbHtrA oligonucleotides (Figure S1). A similar PCR amplification on BeWo cells using the same oligonucleotide primers pair demonstrated the lack of amplification of the human HtrA (HuHtrA), confirming the specificity of these primers for the bacterial CbHtrA. Next, the CbHtrA mRNA expression was analyzed by qRT-PCR performed on bacterium-free BeWo cells or BeWo cells infected with *C. burnetii* and evaluated using the primers specific to the bacterial CbHtrA. As shown in Figure 2A, a CbHtrA mRNA was expressed in BeWo cells infected either by the live Nine Mile (NM) or Guiana (Gui) strains of *C. burnetii.* It is worth noting that the CbHtrA expression (Figure 2B) was significantly higher in the avirulent phase II bacteria than in the virulent phase I of the NM and Gui strains of *C. burnetii*.

This result indicates that the CbHtrA mRNA is expressed in *C. burnetii*-infected cells and suggests an association between CbHtrA expression and virulence.

### 2.4. Effects of CbHtrA from C. burnetii on E-Cadherin Expression in BeWo Cells

**Figure 3:**
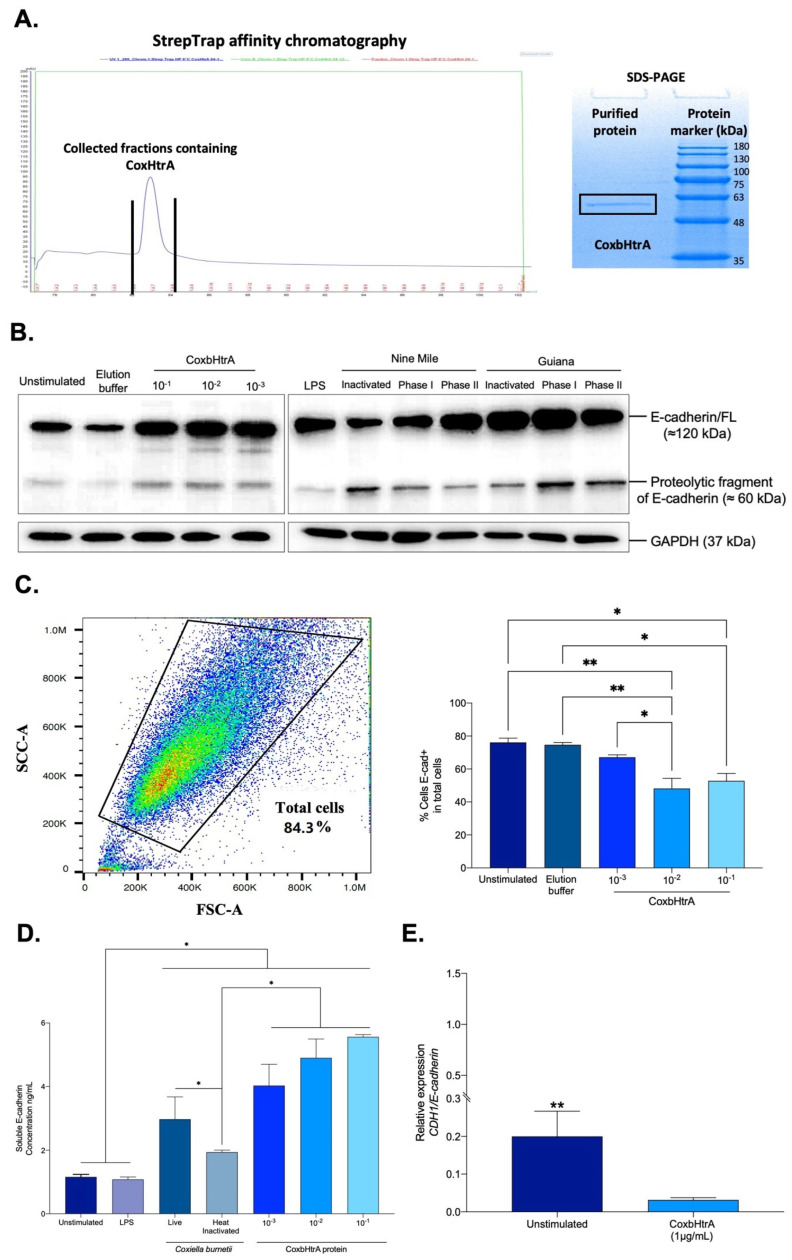
E−cad modulation following a cellular infection by *C. burnetii* or exposure to the recombinant CoxbHtrA protein. (**A**) The tagged recombinant CoxbHtrA protein was purified from transformed *E. coli*. MALDI-TOF (left panel) SDS-PAGE (right panel). (**B**) The quantification by immunoblot of E-cad expression and GAPDH (control) in protein lysates of BeWo cells stimulated with different dilutions of the CoxbHtrA protein and the elution buffer as control. In addition, BeWo stimulated with *E. coli* LPS cells or infected with *C. burnetii* NMI laboratory strain and Guiana strain. (**C**) Flow cytometry analysis of E-cad expression at the surface of BeWo cells (n = 3). Cells were incubated with recombinant CoxbHtrA (dilution 1:10, 1:100, and 1:1000) or were maintained in a cultured medium without additives (unstimulated or incubated, with an elution buffer of the recombinant protein at 1:10 dilution used as a control). The histogram indicates the percentage of cells expressing E-cad under the different experimental conditions. (**D**) The ELISA quantification of sE-cad released in the supernatant of BeWo cells under different experimental conditions (n = 3, unstimulated, *E. coli* LPS-stimulated, inactivated, or infected with live *C. burnetii* Nine Mile strain). (**E**) Relative expression (RE = 2^−ΔCt^), where ΔCt = Ct_Target_ − Ct _Actin_ of CDH1/E-Cad mRNA in BeWo cells untreated or treated with the CoxbHtrA recombinant protein. The non-parametric Mann–Whitney test was used for statistical analysis of all data. For *p*-value < 0.05: symbol *; *p*-value < 0.01: symbol **.

Due to the fact that both CbHtrA and HuHtrA mRNAs can likely be produced in BeWo cells infected by *C. burnetii*, further proof that CbHtrA was functional and competent for cleavage of E-cad was needed. To further explore the functional properties of CbHtrA, we designed a synthetic Coxb-HtrA gene mimicking the CbHtrA sequence. We cloned the full-length putative *htrA* gene sequence into the pET-22b (+) bacterial vector optimized for expression in the electrocompetent BL21(DE3) *E. coli* strain. The expressed protein was purified using an ÄKTA avant system equipped with a StrepTrap HP column. Protein expression was assessed by SDS-PAGE, which confirmed the presence of a protein migrating at the expected molecular weight (about 50 kDa), and this was confirmed by performing MALDI-TOF MS analysis on previously obtained gel bands (Figure 3A). The purified recombinant protein was then tested to evaluate its protease activity. BeWo cells were incubated for 24 h with recombinant CoxbHtrA protein at different dilutions (1:10; 1:100; 1:1000) from the concentration of 1.6 mg/mL, and the cleavage of E-cad was quantified by SDS-PAGE and immunoblot. In cell lysate from BeWo cells exposed to CoxbHtrA treatment, there was an increase in a 60 kDa proteolytic fragment of E-cad (Figure 3B). Unexpectedly, we observed a parallel overproduction in the full-length form of E-cad. Furthermore, the same cleavage profile was observed in BeWo cells infected with the bacterium. To further explore these results, the expression level of E-cad protein on the surface of BeWo cells exposed to the CoxbHtrA protein was measured using an antibody targeting the ectodomain portion of E-cad. Thus, the percentage of BeWo cells expressing membrane-bound E-cad was measured, and a significant decrease in E-cad expression was found in the CoxbHtrA-protein-stimulated cells compared to the unstimulated cells used as controls or cells exposed to the elution buffer of the recombinant protein (Figure 3C). Furthermore, the culture supernatant of BeWo cells exposed to CoxbHtrA treatment was tested by ELISA for the presence of sE-cad released from cells. Live *C. burnetii* induced a higher level of sE-cad than unstimulated cells, cells stimulated with *E. coli* LPS, or cells exposed to heat-inactivated *C. burnetii* (Figure 3D). In addition, when cells were exposed to CoxbHtrA, the sE-cad concentration in cell culture supernatants was significantly higher than in all other experimental conditions with a dose-dependent manner, indicating that CoxbHtrA is a functional sheddase.

These results indicate that *C. burnetii* secretes its own HtrA and that this sheddase is able to generate a cleavage of the integral E-cad. Moreover, incubation of BeWo cells with CoxbHtrA results in a significant decrease of the CDH1/E-cad gene expression (Figure 3E), suggesting the possible activation of a negative feedback loop on the CDH1/E-cad gene expression.

### 2.5. C. burnetii Infection of BeWo Cells Also Modulates the Expression of Human Sheddases

Since we had demonstrated the capacity of CoxbHtrA to function as a sheddase with an ability to cleave the integral membrane E-cad and because several human sheddases (including members of the MMP and ADAM family of sheddases) were previously reported in the literature as being overexpressed in patients with Q fever disease, it was tempting to question whether or not *C. burnetii* could modulate the activity of human sheddases.

**Figure 4:**
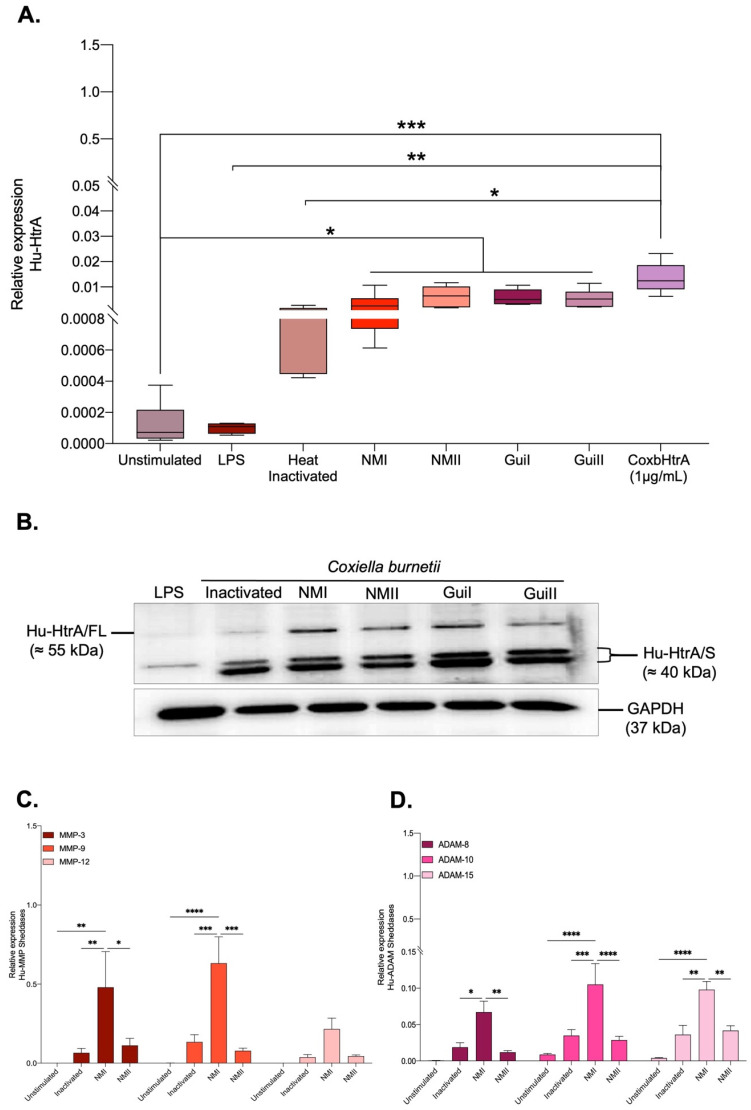
Expression of human sheddases in BeWo cells infected by *C. burnetii.* (**A**) The expression of HuHtrA mRNA in BeWo cells exposed to LPS from *E. coli* and heat-inactivated Guiana *C. burnetii* strain and BeWo cells infected by either phase I or phase II of Nine Mile or Guiana strain of *C. burnetii* was evaluated by qRT-PCR using primers specific to the HuHtrA. (**B**) Quantification by immunoblot of HuHtrA expression and GAPDH (control) in protein lysates of BeWo cells. The HuHtrA expression was analyzed using an anti-HuHtrA antibody for different experimental conditions in which BeWo cells were incubated, with different dilutions of CoxbHtrA protein or the elution buffer as a control (left panel). The HuHtrA was also analyzed for BeWo cells incubated with E. coli LPS or for cells infected with either phase I or phase II of *C. burnetii* NM and Guiana strains (right panel). FL, full length; S, short length. (**C**,**D**) Expression of human sheddases MMP-3, MMP-9, MMP-12, ADAM-8, ADAM-10, and ADAM-15 in BeWo cells infected by *C. burnetii* (n = 6). The analysis of human sheddase gene expressions in BeWo cells infected by the Nine Mile strain of *C. burnetii* either in the virulent phase I (NMI) or avirulent phase II (NMII) was performed using qRT-PCR. The control consisted of bacteria-free BeWo cells and BeWo cells exposed to heat-inactivated NM bacteria. The expression level of investigated genes is illustrated as ± SEM of the relative expression (RE = 2^−ΔCt^), where ΔCt = Ct_Target_ − Ct _Actin_, and compared using the Mann–Whitney U-test. For *p*-value < 0.05: symbol *; *p*-value < 0.01: symbol **; *p*-value < 0.001: symbol ***; *p*-value < 0.0001: symbol ****.

First, the HuHtrA mRNA expression was evaluated using another set of primers specific to the human HtrA. As shown in Figure 4A, the expression of HuHtrA mRNA was significantly increased in all infected conditions compared to unstimulated and LPS stimulated cells, suggesting that the infectious process, besides being associated with the production of CbHtrA, also induces HuHtrA gene expression. It should be emphasized that HuHtrA was also found to be induced when BeWo cells were exposed to heat-inactivated *C. burnetii* and when the cells were incubated with the CoxbHtrA recombinant protein, indicating that infection is not required to activate the expression of HuHtrA but is achieved under bacterial antigen contact or cleavage of E-cad. As shown in Figure 4B, when BeWo cells are infected with *C. burnetii*, the immunoblotting detection of the HuHtrA molecule provides evidence that a new isoform of HuHtrA called HuHtrA/S is overproduced by the BeWo cells. The expression of human sheddases MMP-3, MMP-9, MMP-12, ADAM-8, ADAM-10, and ADAM-15 in BeWo cells infected by *C. burnetii* was also studied. As shown in Figure 4C, we found that the virulent (phase I) Nine Mile strain induces MMP-3 and MMP-9 expression and, at a lower level, an expression of ADAM-10 and ADAM-15. This expression seems to be associated with the virulence since infection with the avirulent (phase II) Nine Mile does not induce a similar activation of human sheddase gene expression.

These results indicate that during infection of BeWo cells by *C. burnetii,* both prokaryotic (CbHtrA) and eukaryotic (HuHtrA) sheddases are induced, with a likely dominance of CbHtrA expression during the avirulent phase II.

### 2.6. Evaluation of the Role of CbHtrA in the Physiopathology of C. burnetii Infection

**Figure 5:**
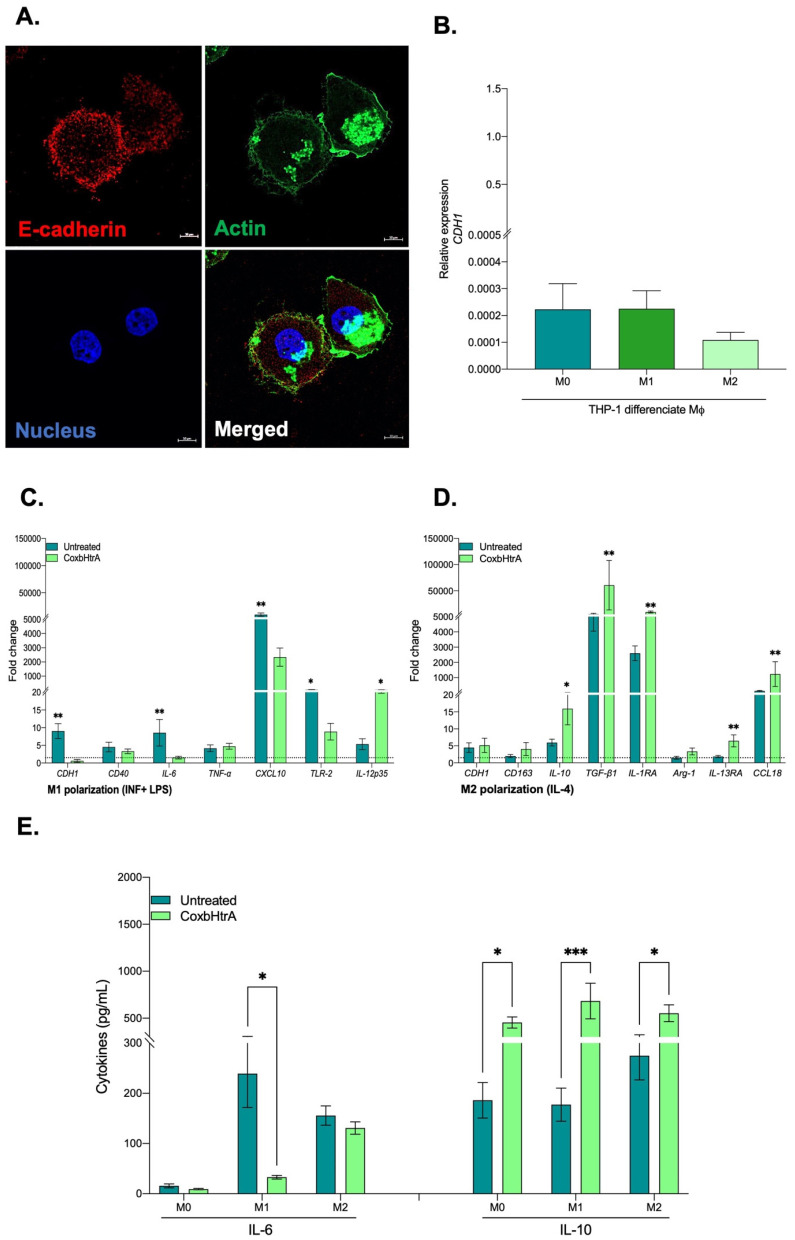
Expression of human E-cad in macrophage-differentiated THP-1. (**A**) Illustration of single-plane confocal microscope analysis of E-cad in THP-1 macrophage. Actin expression was shown as the control as well as the labeling of the nucleus. Scale bar: 10 μm. (**B**) CDH1/E-cad mRNA expression in THP1 macrophages polarized by treatment with IFN-γ and lipopolysaccharide for the M1 phenotype, IL-4 for the M2 phenotype, or without agonist for the M0 phenotype. The results (n = 6) are expressed as RE, where RE = 2^−ΔCt^. (**C**,**D**) mRNA expression patterns of different cytokines in THP-1 macrophages (M1 and M2 phenotypes) untreated or treated with CoxbHtrA for 24 h. The results (n = 6) are expressed as fold change (FC = 2^(−ΔΔCt)^, where ΔΔCt = [(Ct_Target_ − Ct_Actin_) treated] − [(Ct_Target_ − Ct_Actin_) untreated]. Gene expression was considered modulated when the fold change was ≥1.5 (indicated by the dotted line). (**E**) The release of IL-6 and IL-10 by THP-1 macrophages (n = 3) polarized into M1 or M2 phenotypes or unpolarized (M0) and untreated or treated with CoxHtrA was determined by immunoassay. The data represent a mean ± standard error. Statistical analyses were performed using the Mann–Whitney U-test (untreated vs. CoxbHtrA treatment). For *p*-value < 0.05: symbol *; *p*-value < 0.01: symbol **; *p*-value < 0.001: symbol ***.

Although the trophoblastic BeWo cell in vitro model was well suited for the functional characterization of CbHtrA and CoxbHtrA, it cannot be considered relevant for investigating the pathophysiology of *C. burnetii* infections. Indeed, monocytes and macrophages are the main targets for *C. burnetii* during infection of humans with the bacteria. More precisely, it was previously reported that the monocytes in which *C. burnetii* survives without replication are the classically activated macrophages (CAMs or M1), which exhibit a proinflammatory M1-type response (e.g., TNF, IL-1β, IL-6, IL-12, and NO production), whereas macrophages in which *C. burnetii* slowly replicates are polarized towards an anti-inflammatory M2-type response [36] (e.g., TGF-β1, IL-4, IL-6, IL-10, and arginase 1). To study the existence of a possible association between the production of CbHtrA and an effect on the cellular targets of the bacterium, a model of THP-1 cells—differentiated into macrophages, which express high levels of E-cad at their surface (Figure 5A) and can be induced towards an M1 or M2 polarization—were exposed to the active CoxbHtrA sheddase and analyzed for an M1/M2 mRNA interleukins signature. To further confirm the functional potential of CoxbHtrA, THP-1-macrophages (M0, M1, or M2 types) were analyzed for cell-surface E-cad expression. As shown in Figure 5B, the population expressing the lowest levels of E-cad were the M2-like-type cells, suggesting an association between down regulation E-cad (either at the transcriptional level, protein turn over, or shedding) with M2-type differentiation. Next, THP-1 cells polarized into M1 type or M2 type were tested for the expression of several genes either in the presence or absence of active CoxbHtrA sheddase. As shown in Figure 5C,D, treatment with CoxbHtrA triggers a significant down-regulation of most genes, including IL-6, which is a representative cytokine of the M1 phenotype and a significant up-regulation of genes such as the IL-10, the expression of which defines the M2-type cells.

**Figure 6:**
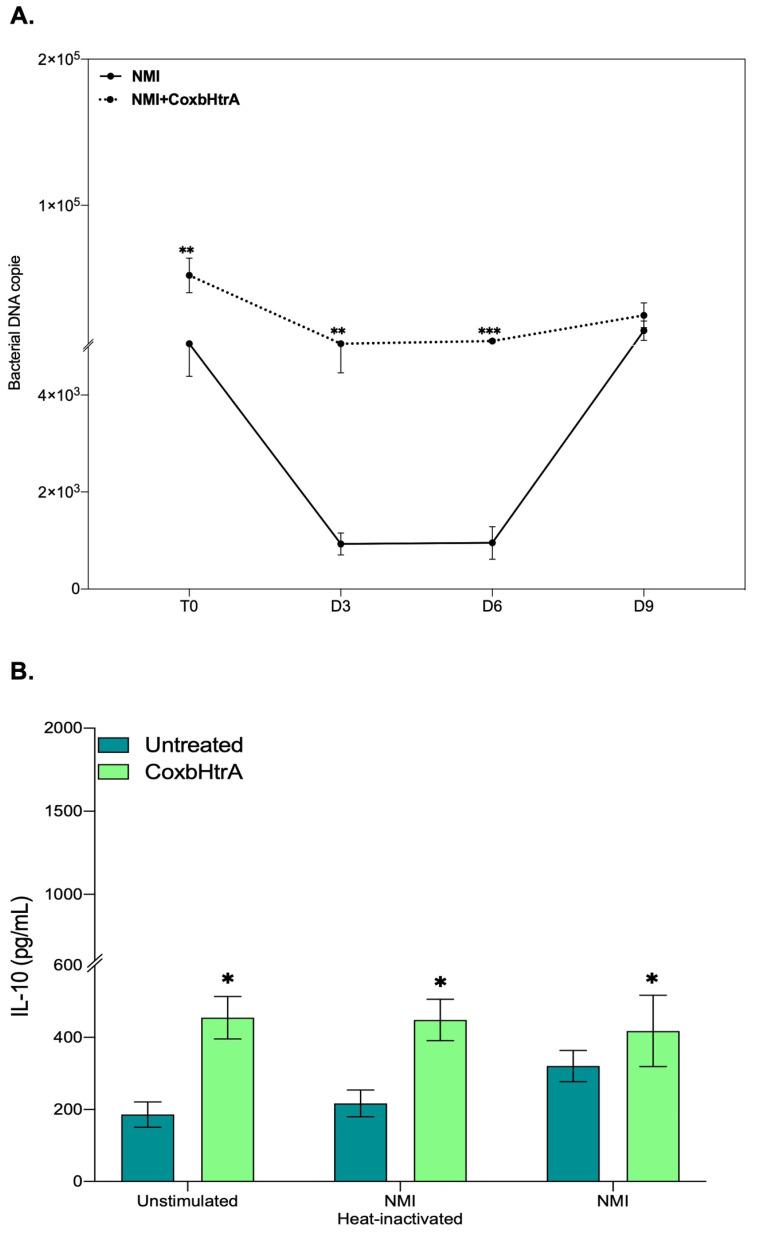
Role of CoxbHtrA in THP-1-macrophages’ response to *C. burnetii*. Macrophages differentiated from THP-1 cells were untreated or treated with CoxbHtrA for 24 h before infection with *C. burnetii* (50 bacteria per cell). (**A**) For *C. burnetii* persistence, THP-1-macrophage cells (n = 8) were infected for 4 h: a period designed as the uptake phase (or T0). After the bacterial uptake phase, the cells were cultured for 9 days, and the presence of bacterial DNA copies was assessed every 3 days by qPCR (in duplicate). (**B**) The release of IL-10 by THP-1 macrophage (n = 3) with or without pretreatment and infected by live *C. burnetii* or exposed to heat-inactivated bacteria for 24 h was determined by immunoassays. Data represent mean ± standard error. Statistical analyses were performed using the Mann–Whitney U-test (untreated vs. CoxbHtrA treatment). For *p*-value < 0.05: symbol *; *p*-value < 0.01: symbol **; *p*-value < 0.001: symbol ***.

Since previous publications reported that *C. burnetii* replication occurs in monocytes from patients with Q fever endocarditis who overproduce the IL-10 immunoregulatory cytokine that enables the bacteria to survive in “disarmed” monocytes [37,38], we aimed to test what the impact of active CoxbHtrA sheddase treatment on the replication of virulent *C. burnetii* could be. Under CoxbHtrA treatment, the bacterial DNA copy number is significantly increased on day 3 and day 6 post infection compared to untreated cells (Figure 6A), suggesting that the CoxbHtrA treatment “disarms” the cells, allowing the bacteria to bypass the latency phase seen in the control experiment. This is consistent with the observation that 24 h after CoxbHtrA treatment, the cells infected with the virulent *C. burnetii* show an increased transcription of the IL-10 mRNA and other mRNA such as TGFβ, indicating a preferential M2 signature (Figure S2). This overexpression of IL-10 mRNA is confirmed at the protein level (Figure 6B). Moreover, as expected, this IL-10 production is associated with a down-regulation of E-cad expression (Figure S3).

## 3. Discussion

Thus far, many results previously published in the literature have argued in favor of an activation of MMP metalloproteinase and ADAM family of human sheddases during *C. burnetii* infections. The activation of such sheddases in human cells could provide a rational hypothesis to explain why we found high concentrations of sE-cad in sera of Q fever patients and why infection of BeWo cells by *C. burnetii* leads to the modulation of the E-cad/β-cat pathway [27,31]. However, apart from the human sheddases that were likely to contribute to E-cad proteolysis and sE-cad release in Q fever patients, we also wanted to explore the possibility of a direct mechanism of cleavage initiated by the bacterium itself, assuming the possible synthesis of a sheddase encoded in the genome of *C. burnetii.*

### 3.1. The Genome of C. burnetii Contains a Gene Encoding an HtrA Sheddase

Several bacteria have previously been found to be able to encode sheddases that cleave E-cad [28]. In light of this observation, it was possible to postulate that *C. burnetii*, like many other bacteria species, encodes a sheddase or sheddases in its genome. To improve our knowledge on putative sheddases produced by *C. burnetii* itself, we used in silico analysis for an initial screening. Among the hits, we found hypothetical proteins named CbMMP-3-like and CbMMP-9-like, presenting short-sequence identity with HuMMP3 and HuMMP9. The blasting search in the *NCBI* database revealed that the CbMMP-3-like sequence did indeed correspond to the nuclear transport factor 2 protein and that the CbMMP-9-like sequence corresponded to the L28 protein of the large 50S ribosomal subunit of *C. burnetii.* In bacteria, the L28 protein has been shown to be encoded by the rpmB operon and required for ribosome assembly in association with the L33 protein [39]. Because the nuclear transport factor 2 protein showed unexpected similarities with HuMMP-3 in the region that correspond to the catalytic domain of HuMMP-3 and shared some similarities with the BFT toxin bacterial sheddase, we performed a reverse BLAST that led us to the conclusion that it is very unlikely that such a molecule might exhibit sheddase activity. Using the same approach, we also eliminated the L28 protein and the CbADAM-15-like as possible candidate for sheddase activity. Two additional hypothetical *C. burnetii* proteins with either a Rif1 or OmpA domain emerged from the in silico screening. However, none of those molecules met the criteria for a possible sheddase. Among the bacterial sheddases used in this study to search hits in *C. burnetii*, only one, HtrA [32], turned out to be of high interest because the CbHtrA-like sequence of RSA 493 Nine Mile, NL3262 Netherland, and Z3055 strains were identical beyond the 451 amino acid residues and because of the probable capacity for secretion. The hypothetical CbHtrA exhibited high sequence identity with the HtrA from bacteria such as *S. typhimurium*, enteropathogenic *E. coli*, and *S. flexneri*, with a conserved secondary structure organization that included a signal peptide, a serine protease domain, and two PDZ domains. Within the serine protease domain, the catalytic triad of H, D, and S [33,40] was conserved. To determine if this protein could be responsible for the cleavage of E-cad during *C. burnetii* infection, cells were infected in vitro with *C. burnetii*, and the expression of the CbHtrA gene was evaluated, which turned out to be expressed. Furthermore, a recombinant CoxbHtrA protein was found to act as a sheddase, as it was able to trigger sE-Cad shedding when incubated with BeWo cells that express E-Cad at their surface.

### 3.2. How Could C. burnetii HtrA Sheddase Act to Cleave E-Cad When the Bacterium Is inside a Cell?

Although we demonstrate here that the HtrA sheddase of *C. burnetii* can be functionally active in vitro, many questions remain to be addressed. The purified CoxbHtrA protein used in our study was obtained after lysis of competent BL21(DE3) expressing the recombinant protein. Therefore, the question must be asked whether this is physiological and whether the CbHtrA sheddase produced by the *C. burnetii* can actually be produced in the extracellular environment to cleave E-cad on the cell surface. *C. burnetii* is a Gram-negative, strictly intracellular bacterium; therefore, the mechanism by which this protease cleaves the E-cad expressed at the cell membrane remains obscure. Protein trafficking across the bacterial envelope is a complex process that can possibly influence the signaling and integrity of the host cell. The bacterium uses a number of nanomachines (protein secretion system of various types) to allow protein trafficking and export across membranes [41]. Pathogenic Gram-negative bacteria secrete effector proteins into the periplasm, outer membrane, or external milieu by different secretion pathways [42,43]. In light of the other bacterial models in which the bacterium produces an HtrA, the hypothesis of secretion by transport vesicles could be put forward. Similarly, it is possible to imagine that in the bacterial population, certain bacteria could lyse and thus directly release the soluble HtrA into the extracellular environment. In Gram-negative bacteria (including *Shigella* and pathogenic *E. coli*), there is a growing family of serine proteases secreted to the external milieu by a secretion mechanism called an autotransporter pathway [44,45]. In the model of *C. jejuni*, it was demonstrated that HtrA is expressed in the periplasmic space, but it can also be secreted into the extracellular environment and cleave the E-cad adherens junctions [46,47,48]. HtrAs form oligomers, and these proteases are present in two functional states: a resting state (inactive) and an active state, whose tertiary and quaternary structures differ [49]. Assembly into oligomers is transient, and the proteins return to the resting state as soon as the substrate is depleted [36,50]. Interestingly, *Chlamydia trachomatis*, an obligate intracellular human pathogen, as is *C. burnetii*, produces HtrA in the periplasm through a sec-dependent pathway. HtrA is also exported outside via an outer membrane vesicle mechanism and is found in the host cell cytosol [51,52,53]. In addition, it was reported that *Glaesserella* (*Haemophilus*) *parasuis* infection activates the canonical Wnt/β-catenin signaling pathway, leading to the disruption of the epithelial barrier, with a sharp degradation of E-cadherin and an increase of the epithelial cell monolayer permeability [54], which is an observation very similar to what we recently reported with *C. burnetii* [31].

### 3.3. C. burnetii Induces a Complexe Profile of Sheddases Expression during the Virulent and Avirulent Phases of Infection

We have also evidenced that *C. burnetii* infection induces HuHtrA and that the level of induction differs with respect to the virulent phase I or avirulent phase II. Thus far, it is not clear why such variations are observed or what the respective influence of CbHtrA and HuHtrA is on the cleavage of E-cad during *C. burnetii* infection. In this paper, we also studied the activation of MMP (MMP-3, MMP-9, and MMP-12) and ADAM (ADAM-8, ADAM-10, and ADAM-15) gene expression in BeWo cells infected by *C. burnetii*. We found that the virulent (phase I) Nine Mile strain induces MMP-3, MMP-9 expression, and, at a lower level, an expression of ADAM-10 and ADAM-15. Infection with the avirulent (phase II) Nine Mile does not show such drastic induction of human sheddases. The overexpression of MMP molecules was previously reported in Q fever patients [29] or after cells had been exposed in vitro to *C. burnetii* [30]. MMP are zinc-dependent proteases [55,56]. The zymogen of MMP-3 (proMMP-3), a 54kDa protein, is activated in the 45kDa and 23kDa active forms by limited proteolysis catalyzed by elastase and cathepsin G [57]. The active form of MMP-3 was reported to be overexpressed in breast cancer and found to catalyze E-cad proteolysis in that cancer [58,59]. MMP-3 is an activator of the proMMP-9 [60]. The proMMP-9 is secreted as a 92kDa. The active MMP-9 enzyme is an 82kDa gelatinase that readily digests gelatin [61]. Zona occludens 1, α1-Antiproteinase, latent TGF-β1, latent VEGF, fibrin, and NG2 proteoglycan are also substrates of MMP-9 [62]. Interestingly, MMP-9 was also identified as a key gene in mantle cell lymphoma [63], a non-Hodgkin’s lymphoma. An overexpression of human MMP-3 in dendritic cells (DC) infected in vitro by *C. burnetii* and variations in the HuMMP-9 expression in human cells exposed in vitro to heat-inactivated *C. burnetii* was also observed using a screening of differential expressions of 45,000 genes by microarray analysis. It is possible that the overexpression of MMP-3 modulates MMP-9 expression in cells infected by *C. burnetii* [60], thereby contributing to the physiopathology of persistent Q fever. MMP-9 overexpression was previously reported following bacterial lipopolysaccharide (LPS) stimulation of lung alveolar macrophages [64]. In addition, an overexpression of human HuADAM-15 in persistent Q fever patients’ blood cells and variations in the HuADAM-10 expression in human cells exposed in vitro to heat-inactivated *C. burnetii* were also observed using our microarray approach. The HuADAM-15 contains a signal peptide and a metalloproteinase domain, followed by a disintegrin-like domain, cysteine-rich domain, epidermal growth factor domain, short connecting linker, hydrophobic transmembrane segment, and cytoplasmic tail. The protein contains a functional catalytic consensus sequence (HEXGEHXXGXXH). ADAM-15 has been linked to a number of different cancerous diseases [65] as well as to the modulation of epithelial cell-tumor cell interactions [66]. It was reported in the literature that the ectodomain shedding of E-cad by ADAM-15 supports the ErbB receptor activation associated with the progression of prostate and breast cancer [67]. Altogether, these data indicate that *C. burnetii* infection is associated with the modulation of several sheddases belonging to the MMPs and/or ADAM families and that qualitative and/or quantitative individual variations can be evidenced from patient to patient during *C. burnetii* infections. It is also worth noting that when human sheddases are highly expressed in association with *C. burnetii* infection (e.g., Nine Mile phase I), the CbHtrA is not highly expressed, while in Nine Mile phase II infection, the CbHtrA is highly expressed, and the HuMMP and HuADAM are less expressed, suggesting the existence of a molecular mechanism involved in regulating the balance between the prokaryotic and eukaryotic sheddase gene expression.

### 3.4. What Could Be the Consequences of the Expression of the HtrA Sheddase in the Intracellular Cycle of Replication of C. burnetii and the Pathophysiology of Q Fever?

Although the complete pattern of molecular crosstalk between *C. burnetii* and its target cell leading to the shedding of soluble E-cad could be more complex than a simple, direct action of CbHtrA (with concurrent expression of HuHtrA and likely other human sheddases), our data provide the first direct evidence that *C. burnetii* encodes its own CbHtrA sheddase that is able to cleave E-Cad. This is also the first description of a functional HtrA sheddase encoded in the *C. burnetii* genome of four different strains of this bacteria. Our data suggest that *C. burnetii* possesses a proteolysis strategy to manipulate host cell signaling pathways via the secretion of HtrA, which may play an important role in interactions with host cells and bacterial virulence. Moreover, this work clearly establishes a link between the presence of gene coding for a functional HtrA sheddase (CbHtrA) in the genome of *C*. *burnetii*, with the ability of this gene to encode a functional sheddase (CoxbHtrA) that cleaves the E-cad, leading to an M2 polarization of the target cells and inducing their secretion of IL-10. Intracellular bacteria replication is likely controlled by the levels of IL-10, an immunoregulatory cytokine that is known to inhibit the production of reactive oxygen and reactive nitrogen intermediates, thereby preventing the generation of toxic compounds by these intracellular bacteria [68]. It was previously reported that *C. burnetii* replication occurs in monocytes from patients with Q fever endocarditis who overproduce IL-10, while the bacteria are killed in monocytes from patients with acute Q fever, suggesting that IL-10 enables monocytes to support *C. burnetii* replication and favors the development of chronic Q fever [38]. Here, we demonstrate that the recombinant active CoxbHtrA sheddase triggers a massive induction of IL-10 in THP-1 cells, which is characteristic of an M2-type response, and we found that the lowest levels of E-cad expression were in the M2-like-type cells, suggesting an association between down-regulation E-cad with M2-type differentiation and the induction of IL-10 production. Under CoxbHtrA treatment, the NMI virulent bacterial DNA copy number is significantly increased on day 3 and day 6 post infection compared to untreated cells, suggesting that bacterial replication is boosted through the action of the CoxbHtrA sheddase. For the first time, these results partially lift the veil on the mechanism that enables the bacteria to replicate intracellularly. These results open new research avenues to study the complexity of the clinical aspects of Q fever.

## 4. Materials and Methods

### 4.1. Coxiella Burnetii

Two laboratory strains of *Coxiella burnetii* were used in this study: the virulent Nine Mile I (NMI) and Guiana I (GuiI) and the isogenic Nine Mile and Guiana avirulent or “phase II” strain (NMII and GuiII) generated through serial in vitro passage, during which it undergoes a chromosomal deletion affecting the LPS biosynthesis. NMII and GuiII LPS lack the unique branched terminal sugar-containing O-polysaccharide chain that is characteristic of the virulent strain [69,70]. *Coxiella burnetii* (Nine Mile strain RSA496, Guiana Strain Cb175) stocks were used to infect L929 cells. The L929 cells infected with either the Nine Mile or Guiana strain of *C. burnetii*, cultured with a Minimum Eagle Medium (MEM, Invitrogen, Waltham, MA, USA) supplemented with 4% Fetal Bovine Serum (FBS, Invitrogen, Waltham, MA, USA) and 1% 2 mM L-Glutamine (Invitrogen, Waltham, MA, USA), were incubated at 35 °C in a 5% CO_2_ atmosphere for the in vitro production of the bacteria as previously described [71]. After two and five passages, respectively, for the phase I and phase II, the infected cells were sonicated, and the cell-free supernatants were centrifuged to harvest the bacteria, which were then washed and stored at –80 °C until they were used. Gimenez staining and qPCR (using the *com-1* gene) were used to estimate the concentration of bacteria in each sample.

### 4.2. In Silico Sequence Analysis

The genomes of four laboratory strains of *C. burnetii* were analyzed. The complete genomic sequences of RSA 493 Nine Mile [72], NL3262 Netherland [73], Z3055 clone [74] genotypically related to the strain causing the Netherlands outbreak, and Cb175 Guiana strain [9] were previously published and deposited in databases. The accession numbers for the genomic sequences of *C. burnetii* are as follows: LK937696 *C* (Z3055 strain); NC_002971.4 *C* (RSA 493 strain); NZ_CP013667.1 (3262 strain); and HG825990.3 (Cb175 strain). The protein-coding genes within these bacterial genomes were predicted using the open-source prodigal (PROkaryotic DYnamic programming Gene-finding ALgorithm) software tool [75].

The predicted coding sequences were converted into amino acid sequences and then subjected to blastp analysis against the 22 known sheddases [25] listed in Table S1. Only blast results with an e-value less than 0.05 were further investigated. Furthermore, we used the SignalP server (https://services.healthtech.dtu.dk/services/SignalP-6.0/, accessed on 11 May 2023) [76] for predicting signal peptides to identify proteins compatible with secretion. The amino acid sequence of the final sheddase candidate gene identified as present in all four strains of *C. burnetii* was subjected to multiple sequence alignment using Clustal Omega, available at https://www.ebi.ac.uk/Tools/msa/clustalo/, accessed on 11 May 2023.

### 4.3. Production of Recombinant CbHtrA

A synthetic CoxbHtrA gene (length 1415 bp) cloned in the NdeI/NotI cloning site of the Novagen pET-22b(+) bacterial vector and optimized for *E. coli* expression was purchased from GenScript Biotech (Leiden, The Netherlands). The expressed protein was designed to include an N-terminal Strep-Tag WSHPQFEK (sequence of the tagged recombinant protein. Competent BL21(DE3) was transformed with the synthetic construct and grown in auto-inducing ZYP-5052 media at 37 °C until it reached an O.D._600nm_ of 0.6; then, the temperature was set to 20 °C for 20 h. After centrifugation (5000× *g*, 30 min, 4 °C), the resulting bacterial pellet was resuspended in 50 mM Tris pH 8 and 300 mM NaCl and stored at –80 °C overnight. The bacterial extract was thawed and incubated on ice for 1 h after adding lysozyme, DNAse I, and PMSF (phenylmethylsulfonyl fluoride) to final concentrations of 0.25 mg/mL, 10 µg/mL, and 0.1 mM, respectively. The lysate was then submitted to sonication on a Q700 sonicator system (QSonica), and cells debris was discarded following a centrifugation step (12,000× *g*, 20 min, 4 °C). The recombinant CoxbHtrA protein was purified using an ÄKTA avant system (GE Healthcare, Chicago, IL, USA) equipped with a 5 mL StrepTrap HP column (GE Healthcare). The wash buffer contained 50 mM Tris pH 8 and 300 mM NaCl, and the elution buffer was 50 mM Tris pH 8, 300 mM NaCl, and 2.5 mM desthiobiotin. Protein expression was assessed by SDS-PAGE and confirmed by performing MALDI-TOF MS analysis on the gel bands previously obtained. The protein concentration was measured using a Nanodrop 2000c spectrophotometer (Thermo Scientific, Waltham, MA, USA).

### 4.4. In Vitro Cell Culture Model and Infection

The human trophoblastic BeWo cell line was purchased from the American-type culture collection (ATCC, CCL-98, Bethesda, MD, USA) and cultured in a Dulbecco’s Modified Eagle Medium F-12 Nutrient Mixture (DMEM F-12, Invitrogen, Waltham, MA, USA) containing 10% Fetal Bovine Serum (FBS) (Invitrogen, Waltham, MA, USA). BeWo cells (2 × 10^5^ cells/well) were cultured in flat-bottom 24-well plates for 12 h and were then infected with the live virulent and avirulent *C. burnetii* Nine Mile (NMI and NMII) strain and Guiana (GuiI and GuiII) strains or were exposed to heat-inactivated virulent *C. burnetii* GuiI strains at a 50:1 bacterium-to-cell ratio or stimulated with 100 ng/mL of lipopolysaccharide (LPS) from *E. coli* (O55:B5; Sigma-Aldrich, Burlington, MA, USA) for 24 h at 37 °C in a 5% CO_2_ atmosphere.

THP-1 cells were cultured in RPMI-1640 containing 10% FBS, 2 mM glutamine, 100 U/mL penicillin, and 50 μg/mL streptomycin and differentiated into macrophages after treatment with 50 ng/mL phorbol-12-myristate-13-acetate (Sigma Aldrich) for 48 h [77,78]. Then, THP-1 cells were polarized for 18 h into the M1 phenotype in the presence of IFN-γ (20 ng/mL) and LPS (100 ng/mL) or into the M2 phenotype using IL-4 (20 ng/mL) or were maintained in the resting state without polarization (M0 phenotype) [79]. The polarization status was confirmed by measuring the expression of M1 and M2 genes (Table S2). As for infection experiments, THP-1 cells (2 × 10^5^ cells/well) were infected with the live virulent and avirulent *C. burnetii* Nine Mile strain (NMI and NMII).

### 4.5. DNA/RNA Extraction and Reverse Transcription Assay

Cellular and bacterial DNA were extracted using a Nucleic Acid purification kit (OMEGA, Norcross, GA, USA) according to the manufacturer’s instructions. The RNA of infected and uninfected cells was extracted using an RNeasy Mini Kit (QIAGEN SA, Les Ulis, France) with a DNase I step according to the manufacturer’s instructions. Starting with 100 ng of purified RNA, the first-strand human cDNA was obtained using oligo(dT) primers and Moloney murine leukemia virus-reverse transcriptase (MMLV-RT kit; Life Technologies, Carlsbad, CA, USA) and the SuperScript™ VILO™ cDNA Synthesis Kit for bacterial cDNA.

### 4.6. Polymerase Chain Reaction (PCR) Standard

The oligonucleotide primers designed (Fwd: 5′ GGCTCCCATTACTCCTGCC 3′; Rev: 5′ GAATCCGAATACCGTTGACAG 3′) for amplification of the CbHtrA gene allowed for amplification of a DNA fragment of 897 bp, corresponding to a portion of the gene in both strains. First, 5 μL of cellular/bacterial DNAs and cDNAs from infected and uninfected cells were used for the outer primer polymerase chain reaction. The 25 μL of polymerase chain reaction of each reaction included 12.5 μL of Master Mix AmpliTaq Gold™ 360 (Applied Biosystems, Woburn, MA, USA), 20 mM of each primer, and 6 μL of DNAse/RNAse-free water. The reaction was carried out in an Applied Biosystems MiniAmp Plus (Applied Biosystems, USA). The polymerase chain reaction was run at 95 °C for 15 min (95 °C for 30 s, 57 °C for 30 s, and 72 °C for 52 s × 35 cycles) at 72 °C for 5 min.

### 4.7. Quantitative Reverse Transcription–Polymerase Chain Reaction (qRT-PCR)

The qRT-PCR experiments were performed using specific oligonucleotide primers and hot-start polymerase (SYBR Green Fast Master Mix; Roche Diagnostics, Mannheim, Germany). The amplification cycles were performed using a C1000 Touch Thermal cycler (Biorad, Hercules, CA, USA). The specific primers used in this study were Human HtrA (*HuHtrA*), *Coxiella burnetii* HtrA (*CbHtrA*), and E-cadherin (*CDH1*). The results were normalized using the housekeeping gene β-actin (*ACTB*) and expressed as the relative expression (RE = 2^-ΔCT^), where ΔCt = Ct_Target_ − Ct_Actin_, and expressed as fold change (FC = 2^(−ΔΔCt)^), where ΔΔCt = [(Ct_Target_ − Ct_Actin_)_stimulated_] − [(Ct_Target_ − Ct_Actin_)_unstimulated_], as previously described [80]. Ct values were defined as the number of cycles for which the fluorescence signals were detected.

### 4.8. Evaluation of Coxb-HtrA Functional Activity

BeWo cells (2 × 10^5^ cells/well) were cultured in flat-bottom 12-well plates for 12 h and were incubated with different dilutions (1:10, 1:100, and 1:1000) of 1.6 mg/mL of CoxbHtrA recombinant protein. The recombinant protein elution buffer (1:10) (50 mM Tris pH 8 and 300 mM NaCl) was used as a control. Additionally, the cells were infected with the live virulent *C. burnetii* Nine Mile and Guiana strains or were exposed to heat-inactivated virulent *C. burnetii* strains at a 50:1 bacterium-to-cell ratio or stimulated with 100 ng/mL of lipopolysaccharide (LPS) from *E. coli* (O55:B5; Sigma-Aldrich, USA). After 24 h of infection and/or incubation in a 5% CO_2_ atmosphere, the culture supernatants were collected, centrifuged at 1000× *g* for 10 min, and stored at –20 °C until use.

Protein quantification was evaluated using a Western blot assay. For that purpose, cells were washed with ice-cold phosphate-buffered saline (PBS 1X) and lysed using the total protein extraction kit (NBP2-37853, Novus Biologicals, Centennial, CO, USA). Then, 10 µg of protein was loaded onto 10% SDS polyacrylamide gels. After their transfer onto a nitrocellulose membrane, the blots were incubated overnight at 4 °C with a saturation solution (5% fat-free milk (FFM), 1X PBS, and 0.3% Tween 20). Blots were then incubated (1:500 dilution) with mouse anti-human E-cad mAb (4A2C7, Life Technologies) and anti-human HtrA (PA523395, Life Technologies). The expression of Glyceradehyde-3-Phosphate dehydrogenase (GADPH) was measured using a mouse anti-human GADPH mAb (1:5000, Abnova, Taipei, Taiwan) as the loading control. Afterwards, the blots were incubated (1:5000 dilution) with a sheep anti-mouse horseradish peroxidase-conjugated antibody (Life Technologies, Carlsbad, CA, USA). The proteins were revealed using an ECL Western Blotting Substrate (Promega, Fitchburg, WI, USA).

### 4.9. Flow Cytometry Assay

BeWo cells (1 × 10^6^ cells/well) were cultured in flat-bottom six-well plates and were then incubated in a 5% CO_2_ atmosphere for 24 h with 1.6 μg/mL of the CoxbHtrA recombinant protein. Cells were exposed to 2 mM EDTA in PBS for 15 min at 4 °C to release the adherent cells from the culture plate. After centrifugation at 500× *g* for 5 min, the pellet was suspended in a FACS buffer (2 mM EDTA, 10% FBS in PBS) and was then incubated (1:1000 dilution) for 1 h with a mouse anti-human E-cad ectodomain antibody (HECD-131700, 1:1000 dilution; Life Technologies). After two washes, cells were incubated with a goat anti-mouse IgG and Alexa Fluor-488 secondary antibody. Fluorescence intensity was measured using a Canto II cytofluorometer (Becton Dickinson/Biosciences, le Pont de Claix, France), and the results were analyzed using a FlowJo V10.7.2 software (Becton Dickinson, Franklin Lakes, NJ, USA).

### 4.10. Confocal Microscopy Analysis

THP-1 macrophage cells were cultured on sterile coverslips in 24-well plates. After fixation with paraformaldehyde (3%), the cells were permeabilized with 0.1% Triton X-100 for three minutes and saturated with 3% BSA-0.1% Tween 20-PBS for 30 min at room temperature. Cells were incubated for one hour at room temperature with a mouse monoclonal anti-E-cadherin (4A2C7, Life Technologies, France) directed against the cytoplasmic domain of E-cad and revealed using an anti-mouse IgG (H+L) secondary antibody (Alexa Fluor 555) (Life Technologies). Then, 4′,6′-diamino-2-fenil-indol (DAPI) (1:2500, Life Technologies) and the phalloidin (Alexa-488) (1:500, Ozyme) were used to stain the nucleus and the filamentous actin, respectively.

### 4.11. Antimicrobial Activity of Macrophage-Differentiated THP-1

THP-1-macrophages (2 × 10^5^ cells/well) were pretreated with CoxbHtrA (1μg/mL) for 24 h before being infected with *C. burnetii* bacterium (bacterium-to-cell ratio of 50:1) for 4 h. After extensive washing to remove free bacteria (time designed as T0), infected cells were cultured for 9 additional days. DNA (50 μL volume) was extracted from the total infected cells/assay every 3 days using DNA Mini Kit (Omega, Norcross, GA, USA). Infection was quantified using 2 μL of DNA and real-time quantitative PCR (qPCR) performed with specific primers targeting the *C. burnetii* com-1 gene [81]. Bacterial uptake and their intracellular fate were expressed as the number of bacterial DNA copies within the THP-1-macrophage.

### 4.12. Immunoassays

The quantity of sE-cad in the supernatants was determined using a specific immunoassay (DCADEO, R&D Systems, Minneapolis, MN, USA) according to the manufacturer’s instructions. The minimal detectable concentration of human sE-cad was 0.313 ng/mL. As for the release of IL-10 and IL-6, they were quantified in cell supernatants using specific immunoassay kits purchased from BD Biosciences. The sensitivity of assays was 15.6 pg/mL and 4.68 pg/mL, respectively.

### 4.13. Statistical Analysis

The statistical analyses of the data were performed using the GraphPad-Prism software (version 9.0). The results are presented as the ± standard error of the mean (SEM). The Mann–Whitney U-test was used for group comparison. A *p*-value < 0.05 was considered statistically significant.

## Figures and Tables

**Table 1:** The % identity relates to the percent of identical and related amino acids with respect to the sequence of the sheddase used for the BLAST. The % coverage refers to the percent identity found with respect to the length of the *C. burnetii* sequence studied. Only three *C. burnetii* hypothetical molecules found to share similarities with rhomboid, subtilase, and HtrA carry secretion signal peptides (SIP).

*C. burnetii* Strain	Protein	Sheddases	% Identity	e-Value	% Coverage	Signal IP	Genomic Position
Cb175	HG825990.3_1452	MMP-3	28	5 × 10^−5^	95	No	1.208.002–1.208.280
	HG825990.3_2153	ADAM_15	30	7 × 10^−4^	53	No	1.793.727–1.793.329
	HG825990.3_823	Subtilase	31	3 × 10^−5^	48	Yes	676.754–676.359
	HG825990.3_51	Rhomboid-1	26	6 × 10^−4^	61	Yes	43.537–42.977
	HG825990 3_847	HtrA	42	2 × 10^−112^	100	Yes	699.374–698.019
NL3262	NZ_CP013667.1_1221	MMP-3	30	5 × 10^−4^	45	No	1.131.415–1.131.011
	NZ_CP013667.1_2102	MMP-9	25	0.001	79	No	1.982.404–1.983.099
	NZ_CP013667.1_1792	Subtilase	31	2 × 10^−5^	48	Yes	1.679.995–1.680.382
	NZ_CP013667.1_230	Rhomboid-1	26	6 × 10^−4^	61	Yes	217.053–217.613
	NZ_CP013667.1_1768	HtrA	42	2 × 10^−112^	100	Yes	1.656.010–1.657.365
RSA 493	NC_002971.4_1204	MMP-3	30	5 × 10^−4^	45	No	1.207.961–1.208.365
	NC_002971.4_271	MMP-9	25	0.001	79	No	258.686–258.513
	NC_002971.4_682	Subtilase	31	3 × 10^−5^	48	Yes	676.626–676.228
	NC_002971.4_45	Rhomboid-1	26	6 × 10^−4^	61	Yes	43.528–42.968
	NC_002971.4_703	HtrA	42	2 × 10^−112^	100	Yes	699.241–697.886
Z3055	LK937696.1_1129	MMP-3	30	5 × 10^−4^	45	No	1.100.606–1.102.408
	LK937696.1_272	MMP-9	25	0.001	79	No	258.788–258.549
	LK937696.1_695	Subtilase	31	2 × 10^−5^	48	Yes	676.641–676.243
	LK937696.1_44	Rhomboid-1	26	6 × 10^−4^	61	Yes	43.525–42.965
	LK937696.1_716	HtrA	42	2 × 10^−112^	100	Yes	699.254–697.899

**Table 2:** Identification of the sheddase-like proteins encoded by the *C. burnetii* genome. Blasting the sequences for homologies with the human proteins of MMP and ADAM families against sequences from the *NCBI* database revealed that the hypothetical CbMMP-3-like sequence corresponded to a nuclear transport factor 2 (NTF2) protein and shared some similarities with the BFT toxin bacterial sheddase, while the hypothetical CbMMP-9-like sequence corresponded to the L28 protein of the large 50S ribosomal subunit of *C. burnetii*. To investigate the relevance of these results, a reverse BLAST analysis was conducted in which the best matches between CbMMP-3 like/NTF2, CbMMP-9like/L28 protein, and CbADAM15-like sequences from *C. burnetii* and the full human genome were investigated to identify the sequences producing the most significant alignments. The best matches for CbMMP-3 like/NTF2 were *Homo sapiens* cadherin 13 (e-value of 0.39; percent identity of 87%; percent coverage of 7%), and *Homo sapiens* RAS p21 protein activator 2 (e-value of 0.39; percent identity of 80%; percent coverage of 8%). The best matches for CbMMP-9 like/L28 protein were *Homo sapiens* Rho GTPase activating protein 20 (e-value of 0.76; percent identity of 83%; percent coverage of 17%) and *Homo sapiens* synthrophin gamma 2 (e-value of 0.76; percent identity of 92%; percent coverage of 11%). The best match for CbADAM-15 like protein was *Homo sapiens* cell division cycle 25 B (e-value of 0.11; percent identity of 93%; percent coverage of 6%). Since no human gene found by the reverse BLAST analysis turned out to be a sheddase family member, it is very unlikely that these proteins from *C. burnetii* show sheddase activity. Using the same strategy, we found that the CbRhomboid-1 corresponds to a protein with an Omp-A domain, and CbSubtilase-like corresponds to a protein with a Rif1 domain. Again, no human genes found by reverse BLAST analysis turned out to be a member of the sheddase. The only hypothetical protein found using our screening strategy, which met the required criteria to possibly be a sheddase, was CbHtrA.

*C. burnetii* Strains	Sheddases	Mean e-Value	Mean % Coverage	Identification
Cb175	ADAM_15	7 × 10^−4^	53	Hypothetical *C. burnetii* protein
NL3262 RSA 493 Z3055	MMP-9	0.001	79	50s ribosomal protein L28
Cb175 NL3262 RSA 493 Z3055	MMP-3	5 × 10^−4^ to 5 × 10^−5^	45 to 95	Membrane of the nuclear transport 2 (NFT2)
	Subtilase	2 × 10^−5^ to 3 × 10^−5^	48	Hypothetical *C. burnetii* protein with a putative Rif-1 domain
	Rhomboid-1	6 × 10^−4^	61	Hypothetical *C. burnetii* protein with a putative OmpA domain
	HtrA	2 × 10^−112^	100	*C. burnetii* HtrA protein

## Data Availability

All the data are available in this manuscript.

## Supplementary Materials

The following supporting information can be downloaded at: https://www.mdpi.com/article/10.3390/ijms241310904/s1.
