# Supplementary material for: Identification and Characterization of an HtrA Sheddase Produced by Coxiella burnetii
Source: Int J Mol Sci. 2023 Jun 30;24(13):10904. doi: 10.3390/ijms241310904 (PMC10342153; doi:10.3390/ijms241310904)

Supplementary data.

Materials and Methods

1. Nucleotide sequence of the Coxiella burnetii HtrA (*CbHtrA*) gene-like.

>CbHtrA gene-like
ATGAAAAAATTATCCAAAATTATTCTCAGCAGTATTTTTGCGGGGCTCCCATTACTCCTGCCCG
TCAGTAGTTACGCTCACTTACCCTCCGCTGTCGAAGGAAAAACAATACCCAGCCTTGCACCAA
TGTTGAATAAGACCACACCGAGCGTCGTCAACATTGCCGTGGAAAAGCTGATTCCTCAAACG
CCAAACCCCTTACAACCCGAAATGGATCAAAACACAGCACCAACGAAAGTCTTAGGCGTAG
GCTCGGGTGTAATCATAGACGCCAAAAAAGGCTATATCGTAACAAATGCTCATGTCGTCAAA
GACCAAAAAATCATGGTGGTGACGCTTAAAGATGGTCGCCGTTATCGAGCGAAAGTCATCGG
AAAAGATGAAGGGTTTGATCTGGCTGTGATTCAAATTCACGCGAACCATTTGACCGCACTTCC
CATCGGAAATTCAGATCAATTAAAAGTGGGTGATTTCGTCGTCGCCGTGGGAAGCCCTTTTGG
CTTAACTCAAACAGTCACTTCCGGCGTCATTAGCGCCTTGAATCGCCAAGAACCGCGTATCGA
TAATTTTCAAAGCTTTATTCAAACCGACGCGCCGATTAATCCCGGCAATTCCGGCGGGGCTTT
AATCGATTTAGAGGGCAAATTAATTGGTATTAATACAGCGATTGTCACCCCGTCCGCGGGAAA
TATCGGCATCGGCTTTGCCATTCCCAGCGACATGGTCAAAAGCGTGGCCGAACAATTAATTAA
ATATGGAAAAGTCGAACGCGGCATGCTCGGCGTAACGGCTCAAAATATTACCCCGGAATTAG
CGGACGCCCTAAATTTAAAACATAACAAAGGAGCGCTGGTAACCAAAGTGGTTGCTGAAAGT
CCAGCGGCTAAAGCCGGGGTTGAGGTGCAGGATATTATTGAATCTGTCAACGGTATTCGGATT
CATAGTTCAGCACAACTCCACAACATGCTCGGGCTGGTGCGTCCAGGAACTAAGATTGAACT
AACCGTATTGCGCGACCATAAGGTTCTGCCTATAAAAACGGAAGTAGCCGATCCTAAAAAAG
TGCTATTGCAACGCGAACTGCCCTTCCTCGGCGGCATGCGTATGCAGAAATTCAACGACCTAG
AGCCCGATGGCACTATTTTGCAAGGTGTTTTAGTTACCGGCGTGGACGATAGCAGCGATGGAG
CGCTCGGCGGGTTAGAGCCCGGCGATATCATTATCAGTGCTAATGGCCAATTAACGCCCACGG
TCGATGAGCTAATGAAAATCGCTGAAGGCAAGCCAAAGGAGTTGTTACTGAAAGTGGCGCGG
GGCGCGGGACAATTATTTTTAGTTATCCAACAATCACAATAA

2. Nucleotide and protein sequences used to generate the recombinant CoxbHtrA protein.

>CoxbHtrA nucleotide sequences
CATATGTGGTCACATCCGCAGTTTGAAAAAGAGAACCTGTATTTCCAGAGCAAGAAACTGAG
CAAAATTATCCTGAGCAGCATTTTTGCAGGTCTGCCGCTGCTGCTGCCGGTTAGCAGCTATGC
ACATCTGCCGAGCGCAGTTGAAGGTAAAACCATTCCGAGCCTGGCACCGATGCTGAATAAAA
CCACACCGAGCGTTGTTAATATTGCCGTGGAAAAACTGATTCCGCAGACACCGAATCCGCTGC
AGCCGGAAATGGATCAGAATACCGCACCGACCAAAGTTTTAGGTGTTGGTAGCGGTGTTATTA
TCGATGCCAAAAAAGGTTATATTGTGACCAATGCGCACGTGGTGAAAGATCAGAAAATTATG
GTTGTGACCCTGAAAGATGGTCGTCGTTATCGTGCAAAAGTGATTGGTAAAGATGAAGGTTTT
GATCTGGCCGTGATTCAGATTCATGCAAATCATCTGACCGCACTGCCGATTGGTAATAGCGAT
CAGCTGAAAGTTGGTGATTTTGTTGTTGCAGTTGGTAGCCCGTTTGGTCTGACCCAGACCGTTA
CCAGCGGTGTGATTAGCGCACTGAATCGTCAAGAACCGCGTATTGATAATTTTCAGAGCTTTA
TTCAGACCGATGCACCGATTAATCCGGGTAATAGTGGTGGTGCACTGATTGATCTGGAAGGTA
AACTGATTGGCATTAATACCGCAATTGTTACCCCGAGTGCAGGTAATATTGGTATTGGTTTTGC
AATTCCGAGCGACATGGTTAAAAGCGTTGCAGAACAGCTGATCAAATATGGTAAAGTTGAAC
GTGGTATGCTGGGTGTTACCGCACAGAATATTACACCGGAACTGGCAGATGCACTGAACCTG
AAACACAATAAAGGTGCACTGGTTACCAAAGTTGTTGCCGAAAGTCCGGCAGCAAAAGCCG
GTGTTGAAGTTCAGGATATTATTGAAAGCGTGAACGGCATTCGTATTCATAGCAGCGCACAGC
TGCATAATATGTTAGGTCTGGTTCGTCCGGGTACAAAAATTGAACTGACCGTTCTGCGTGATC

ATAAAGTTCTGCCGATTAAAACCGAAGTTGCCGATCCGAAAAAAGTGCTGCTGCAGCGTGAA
CTGCCGTTTTTAGGTGGTATGCGTATGCAGAAATTTAACGATCTGGAACCGGATGGCACCATT
CTGCAGGGTGTTCTGGTTACCGGTGTGGATGATAGCAGTGATGGTGCATTAGGTGGTCTGGAA
CCTGGTGATATCATTATTAGCGCAAATGGCCAGCTGACCCCGACAGTTGATGAACTGATGAAA
ATTGCAGAAGGCAAACCGAAAGAACTGCTGTTAAAAGTTGCCCGTGGTGCAGGTCAGCTGTT
TCTGGTGATTCAGCAGAGCCAGTAATAAGCGGCCGC

>CoxbHtrA protein sequences (MW : 49,694 Da)
MWSHPQFEKENLYFQSKKLSKIILSSIFAGLPLLLPVSSYAHLPSAVEGKTIPSLAPMLNKTTPSVVNIA
VEKLIPQTPNPLQPEMDQNTAPTKVLGVGSGVIIDAKKGYIVTNAHVVKDQKIMVVTLKDGRRYRA
KVIGKDEGFDLAVIQIHANHLTALPIGNSDQLKVGDFVVAVGSPFGLTQTVTSGVISALNRQEPRID
NFQSFIQTDAPINPGNSGGALIDLEGKLIGINTAIVTPSAGNIGIGFAIPSDMVKSVAEQLIKYGKVER
GMLGVTAQNITPELADALNLKHNKGALVTKVVAESPAAKAGVEVQDIIESVNGIRIHSSAQLHNM
LGLVRPGTKIELTVLRDHKVLPIKTEVADPKKVLLQRELPFLGGMRMQKFNDLEPDGTILQGVLVTG
VDDSSDGALGGLEPGDIIISANGQLTPTVDELMKIAEGKPKELLLKVARGAGQLFLVIQQSQ

Tables S1. Sheddases proteins and theirs accession numbers obtained from the NCBI/NIH, GenBank and EMBL open international databases.

**Sheddases proteins**		**Accession numbers**
**Zinc-dependent matrix metalloproteases**	Gelatinase/MMP2	NP_001289439.1
	Stromelysine/MMP3	NP_002413.1
	Matrilysin/MMP7	NP_002414.1
	Gelatinase type IV collagenase/MMP9	NP_004985.2
	MMP14	NP_004986.1
**A disintegrin and metalloproteinase**	Adamalysin/ADAM10	NP_001101.1
	ADAM15	NP_997080.1
**PITX1 pituitary homeobox**		NP_002644.4
**Plasminogen**		XP_002938616.2
**KLK7 kallikrein-7 isoform 3**		NP_001230055.1
		NP_644806.1
		NP_005037.1
**Cathepsin**		Aaa35655.1
**Two isoforms of DCST1 E3 ubiquitin protein ligase**		NP_689707.2
		NP_001137159.1
**Three isoforms of EFNA4 ephrin-A4 isoform c precursor**		NP_872632.2
		NP_872631.1
		NP_005218.1
**Bft *b. Fragilis* toxin**		Aab50410
**Chain a subtilase**		2lu1_a
**Rhomboid-1**		Abj97615.1
**Htra Do family serine endopeptidase**		WP_005772880.1

Table S2. Primer nucleotide sequences used for RT-qPCR analysis.

Genes	Primes nucleotide sequences	
	Forward primer (5′-3′)	Reverse primer (5′-3′)
	**M1 genes**	
CD40	GGGTTTTCTGTGTACCCTTCCA	TGGAAGCTGCTTAACTGTCCAT
Il-6	CCAGGAGAAGATTCCAAAGATG	GGAAGGTTCAGGTTGTTTTCTG
CxCL-10	GGAAATCGTGCGTGACATTA	AGGAAGGAAGGCTGGAAGAG
TLR-2	ATTCAGCCAGGAGAGAGAACTG	CTACAACCGCATCGTCAAACT
Il-12p35	GTCACTGCCCGAATTCTGAAAG	TGCAAAGCTTCTGATGGATCCT
TNF-α	AGGAGAAGAGGCTGAGGAACAAG	GAGGGAGAGAAGCAACTACAGACC
Il-1β	CAGCACCTCTCAAGCAGAAAAC	GTTGGGCATTGGTGTAGACAAC
	**M2 genes**	
CD163	CGGTCTCTGTGATTTGTAACCAG	TACTATGCTTTCCCCATCCATC
Il-10	GGGGGTTGAGGTATCAGAGGTAA	GCTCCAAGAGAAAGGCATCTACA
TGF-β1	GACATCAAAAGATAACCACTC	TCTATGACAAGTTCAAGCAGA
Il-1ra	TCTATCACCAGACTTGACACA	CCTAATCACTCTCCTCCTCTTCC
Arg-1	CATGGGCAACCTGTGTCCTT	CGATGTCTTTGGCAGATATGCA
Il-13ra	TAGATGGCCATGAAGAGGATGC	CCAAGACCTAGGGATCACAACC
CCL18	TTAGAAGAGGTGGCCTCCAG	TCACTCCTGGCAGATTCCAC
	**Others used genes**	
CbHtrA	GGCTCCCATTACTCCTGCC	CTAAGACTTTCGTTGGTGCTG
HuHtrA	GAGATCACGTCTGGGAAGTC	GAAAGTGACAGCTGGAATCTC
CDH1	GAAGGTGACAGAGCCTCTGGAT	GATCGGTTACCGTGATCAAAAT
ACTB	CATGCCATCCTGCGTCTGGA	CCGTGGCCATCTCTTGCT CG

Results

Figure S1

The presence of the *CbHtrA* gene in the phase I of Nine Mile (NMI) and Guiana (GuiI) strains of *C. burnetii*. Specific amplification by PCR of a fragment of 897 bp corresponding to the size expected using the oligonucleotide primers chosen. This oligonucleotides pair does not amplify the *HuHtrA* gene.

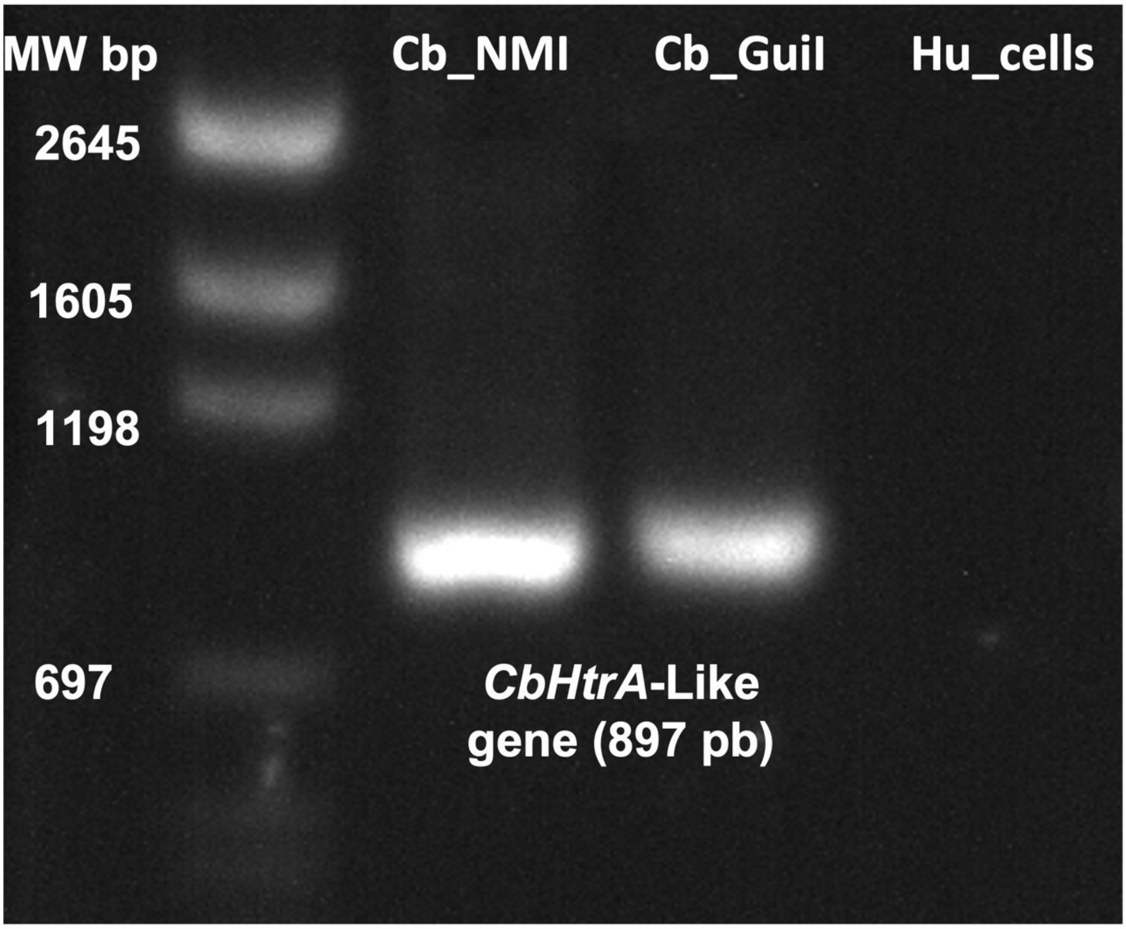

Figure S2

The role of CoxbHtrA in the THP-1-macrophages' response to C. burnetii. Macrophages differentiated from THP-1 cells were treated with CoxbHtrA for 24 hours before infection with C. burnetii (50 bacteria per cell). The expression of THP-1 macrophages (A) M1 and (B) M2 polarization genes was investigated by qRT-PCR. The results (n=6) are expressed as Fold Change (FC = $2^{(-\Delta\Delta Ct)}$ where $\Delta\Delta Ct = [(Ct_{Target} - Ct_{Actin})$treated/infected] - [$(Ct_{Target} - Ct_{Actin})$untreated/uninfected]. Gene expression was considered modulated when the fold change was ≥ 1.5 (indicated by the dotted line). The data represent mean ± standard error. Statistical analyses were performed using the Mann-Whitney U test (untreated vs. CoxbHtrA treatment). For p value <0.05: symbol *; p value <0.01: symbol **.

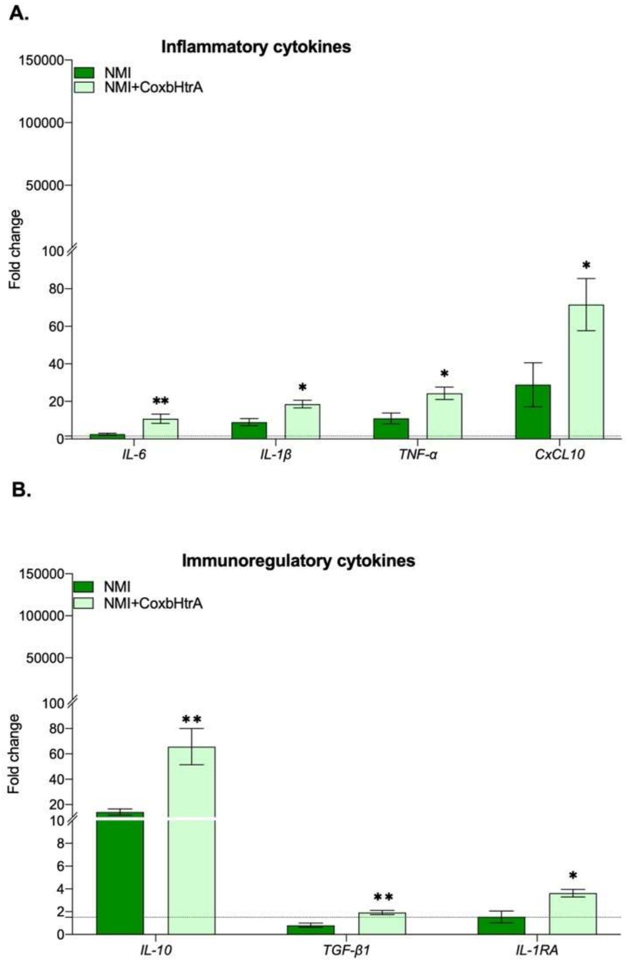

Figure S3

Expression of human CDH1/E-cad mRNA in macrophages differentiated from THP-1 (n=3) with or without pre-treatment, infected by live or heat-inactivated virulent or avirulent *C. burnetii* strains. The results (n=6) are expressed as Fold Change (FC = $2^{(-\Delta\Delta Ct)}$ where $\Delta\Delta Ct = [(Ct_{Target} - Ct_{Actin})treated/infected] - [(Ct_{Target} - Ct_{Actin})untreated/uninfected]$. Gene expression was considered modulated when the fold change was ≥ 1.5 (indicated by the dotted line). The data represent mean ± standard error. Statistical analyses were performed using the Mann-Whitney U test (untreated vs. CoxbHtrA treatment). For p value <0.01: symbol **.

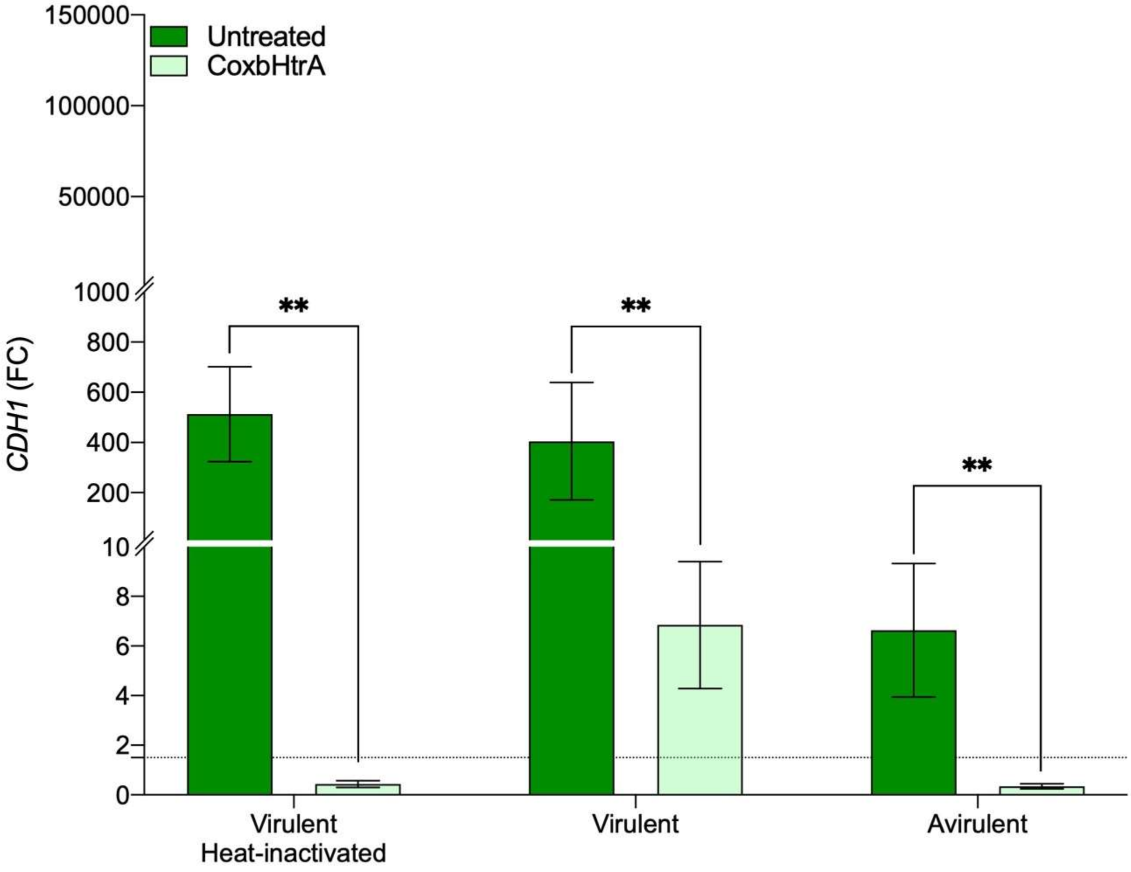

Supplement: Supplementary file 1 [file ijms-24-10904-s001.zip › ijms-2422105-supplementary.pdf]
